# Sources for and quality of neonatal care in 45 low- and middle-income countries

**DOI:** 10.1371/journal.pone.0271490

**Published:** 2022-07-19

**Authors:** Tess Shiras, Sarah E. K. Bradley, Benjamin Johns, Heather Cogswell

**Affiliations:** International Development Division, Abt Associates, Rockville, MD, United States of America; ICMR National Institute of Medical Statistics (ICMR-NIMS), INDIA

## Abstract

Almost half of under-five deaths occur during the neonatal period. Delivery with a skilled attendant, adherence to essential newborn care (ENC) and postnatal care (PNC) standards, and immediate treatment of infections are essential to improve neonatal survival. This article uses Demographic and Health Survey data from 45 low- and middle-income countries to assess 1) levels of ENC and PNC that mothers and newborns receive and how this differs by place of delivery and 2) levels of and sources for care-seeking for neonates sick with fever. For five of the ten ENC and PNC indicators assessed, less than two-thirds of mothers and newborns received care in alignment with global standards. Adherence is higher in private facilities than public facilities for all indicators other than immediate breastfeeding and skin-to-skin contact. Except for immediate breastfeeding, adherence is lowest for newborns born at home with a skilled birth attendant (SBA). Socioeconomic disparities exist in access to skilled delivery and adherence to ENC and PNC, with the largest disparities among newborns delivered at home with a SBA. Private provider adherence to ENC and PNC standards was relatively high for newborns from the wealthiest families, indicating that meeting recommended guidelines is achievable. On average across the 45 countries, half of caregivers for neonates with fever sought care outside the home and 45 percent of those sought care from the private sector. There were substantial socioeconomic disparities in care-seeking for fever, but illness prevalence and sources of care seeking were consistent across wealth quintiles. Closing inequities in neonatal care and care seeking and ensuring that all families, including the poorest, can access high quality maternal and newborn care is crucial to ensure equity and accelerate reductions in neonatal and child mortality.

## Introduction

Despite considerable improvements in child survival over the past 20 years, 2.6 million neonates still die every year in their first month of life [1]. Almost half (47%) of deaths among children under 5 occur during the neonatal period. Further, nearly three-fourths of neonatal deaths occur in the first week of life, and approximately one-third occur on the first day [2]. In 2019, the global neonatal mortality rate was 17 deaths per 1,000 live births [2]. This mortality rate is considerably higher than the Sustainable Development Goal target of 12 neonatal deaths per 1,000 live births, and more than 60 countries are currently projected to miss this neonatal target [3].

Neonatal mortality can be reduced by providing skilled care at birth, quality essential newborn care (ENC), postnatal care (PNC), and immediate treatment for small and sick newborns [3–5]. Levels of facility-based births have increased remarkably in the last two decades, primarily through the public sector, though the private sector plays a key role in many countries [6]. Studies show that immediate breastfeeding and immediate skin-to-skin care are key interventions to reduce neonatal mortality [5,7,8]. The World Health Organization (WHO) postnatal care guidelines recommend that all mothers and newborns stay in the facility for at least 24 hours to ensure that they can receive this essential care [9]. To continue contact with the health system, WHO and the United Nations Children’s Fund (UNICEF) recommend that all neonates receive a postnatal visit from a health professional within the first hour of life and again within the first 24 hours, and research shows that receiving a PNC visit within two days of birth can reduce neonatal mortality by 30 to 60 percent [9–11]. During PNC, it is also critical to ensure that neonates receive recommended postnatal care content—including checking the umbilical cord, assessing the newborn’s temperature, observing breastfeeding, and counselling the mother on breastfeeding [7,12]. Provider adherence to these globally recommended and life-saving standards is a critical component of high quality care for newborns. Understanding gaps in adherence can help country stakeholders to pinpoint areas of weak quality and develop strategies to enhance the quality of ENC and PNC and improve neonatal survival.

Also critical for neonatal survival is early detection of and care seeking for illnesses [13]. In particular, infection including severe bacterial infections is responsible for one-fifth of neonatal deaths [14]. However, caregivers often do not seek care for their sick neonates, seek delayed care, or do not receive high-quality care [10,15,16]. Fever is one of the signs of possible severe bacterial infection (PSBI) and is a tracer indicator frequently measured among neonates in national surveys [17].

The public and private sectors are both important sources for reproductive, maternal, and child health services [18–20], yet few studies have examined the quality of newborn care in the private sector and how it differs from the quality of care provided in the public sector and at home. We could identify only two studies that examined adherence to essential newborn care guidelines in the private sector; one focused on a single country and the other on a single region within a country [21,22]. Understanding how well and where ENC, PNC, and sick neonatal care are provided across countries is critical to catalyzing progress across sectors and achieving high quality neonatal care for all. The goal of this study is to provide insights into the quality of care across the neonatal period and across sectors to help identify gaps and disparities that can inform policy making and program decisions to accelerate reductions in neonatal morbidity and mortality.

## Data and methods

We analyzed data from recent Demographic and Health Surveys (DHS) [23] conducted since 2010 in every country in three regions: East and Southern Asia, East and Southern Africa, West and Central Africa, for a total of 45 countries (Table 1). We excluded surveys from countries outside these three regions because there are too few countries with DHS data in Latin America and the Caribbean, the Middle East and North Africa, Eastern Europe, and Central Asia to reasonably represent those regions. Forty of the 45 surveys are from 2012 or later, though DHS surveys in Burkina Faso, Cameroon, and Mozambique are from 2010 or 2011. DHS surveys are a nationally representative household based survey that use a standard survey questionnaire across all countries where the survey is administered. DHS surveys have large sample sizes, typically between 5,000 to 30,000 households. Additional information on DHS survey methods can be found on the DHS website: https://dhsprogram.com/Methodology/.

**Table 1:** Countries, survey years, and sample sizes included in analysis.

Country and Survey Year	# of newborns delivered in the 2 years preceding the survey (N for Research Question 1)	# of neonates who are <1 month old at the time of survey (N for Research Question 2)
**East and Southern Asia**	**141,785**	**3,108**
Afghanistan 2015	11,762	251
Bangladesh 2014	3,078	71
Cambodia 2014	2,899	59
India 2015–16	97,935	1,839
Indonesia 2017*	6,925	124
Myanmar 2015–16	1,863	33
Maldives 2016–17*	1,168	46
Nepal 2016*	1,970	68
Pakistan 2017–18*	3,872	198
Papua New Guinea 2016–18*	3,605	137
Philippines 2017*	3,871	160
Timor-Leste 2016*	2,837	122
**East and Southern Africa**	**60,173**	**2,251**
Angola 2015–16*	5,837	287
Burundi 2016–17*	5,261	238
Comoros 2012	1,255	28
Ethiopia 2016*	4,081	203
Kenya 2014	7,925	170
Lesotho 2014	1,387	27
Malawi 2015–16*	6,683	265
Mozambique 2011	4,612	99
Namibia 2013	2,051	33
Rwanda 2014–15	3,169	72
South Africa 2016*	1,376	67
Tanzania 2015–16*	4,219	205
Uganda 2016*	5,992	261
Zambia 2018–19*	3,958	201
Zimbabwe 2015*	2,367	95
**West and Central Africa**	**87,771**	**2,872**
Burkina Faso 2010	5,860	147
Benin 2017–18*	5,486	275
Cameroon 2011	4,702	87
Chad 2014–15	6,590	181
Congo (Brazzaville) 2011–12	3,835	72
Cote d’Ivoire 2011–12	3,113	75
Democratic Republic of the Congo 2017–18	7,322	195
Gabon 2012	2,511	72
Gambia 2013	3,481	81
Ghana 2014	2,329	41
Guinea 2018*	3,066	197
Liberia 2013	3,064	64
Mali 2018*	3,926	188
Niger 2012	4,759	131
Nigeria 2018*	12,818	616
Sierra Leone 2013	4,668	129
Senegal 2016–17*	7,459	275
Togo 2017	2,782	46
**All Countries**	**289,729**	**8,231**

*Survey used DHS7 questionnaire.

Our analysis answers the following research questions:

1. What percentage of mothers and newborns receive essential newborn and postnatal care in accordance with global guidelines, and how does the quality of care they receive vary by sector? Further, within the same delivery source category (public sector, private sector, or home with a skilled birth attendant), are there disparities in care by socioeconomic status and/or maternal age?
2. When neonates are sick with fever, what percentage of their caregivers seek advice or treatment outside the home and from where? How do care-seeking patterns for sick newborns compare to those for older infants and children, and are there disparities by socioeconomic status?

To identify disparities in access to and quality of care, we examined all indicators by socioeconomic status using DHS’s asset-based wealth quintiles and compare levels of care sought and received by caregivers and newborns from the poorest 20 percent of the population to the wealthiest 20 percent of the population in each country. In addition, to further investigate equity, we examined all indicators related to the first research question by three categories of maternal age: 15–19, 20–24, and 25–49, to test the hypothesis that adolescent and younger mothers may receive poorer quality care. In addition, we disaggregated all ENC and PNC indicators by babies’ birthweight (<2.5 grams) and by survival status at the time of survey. However, we did not find substantial differences in birthweight or by survival status within the same source category, so we have not included these findings in the results.

To examine how care-seeking patterns may change as children get older, we compared results from neonates in their first month of life in the second research question to several older age categories: young infants 30–59 days, infants 2–11 months, and children 12–59 months. DHS surveys have always measured child age in months, and in recent surveys began measuring child age in days. To standardize across surveys with and without detailed age data, we have defined the first month of life to equal 30 days.

To produce the regional and all-country averages, we pooled data across the 45 DHS surveys and multiplied survey weights by a country-specific constant so that each country contributes equally to regional and all-country estimates. We conducted logistic regressions with robust standard errors to determine if differences in results across sectors, maternal age groups, and socioeconomic status categories are statistically significant, and we only comment on differences when they are significant at the p<0.05 level.

### Data on ENC & PNC

To answer our first research question, we draw on data reported by women about their most recent births and care they and their infant received (or did not receive). We exclude births that occurred more than 2 years before the survey to limit potential recall bias.

Table 2 defines the ENC and PNC indicators analyzed to answer our first research question. Our analysis includes all ENC and PNC indicators collected in the DHS that align with global guidelines for high-quality care for neonates [1,4,8–10].

**Table 2:** ENC and PNC indicators analyzed.

Indicator name and numerator	Indicator denominator*
Essential newborn care indicators	
*Immediate breastfeeding*: # of newborns immediately breastfed	# of newborns delivered vaginally in a facility or at home with a skilled attendant
*Immediate skin-to-skin contact*: # of newborns who received immediate skin-to-skin contact	# of newborns delivered vaginally in a facility or at home with a skilled attendant+
**Postnatal care indicators**	
*24+ hour facility stay*: # of newborns who stayed in the facility for at least 24 hours after delivery	# of newborns delivered vaginally in a facility**
*PNC timing* ➢ # of newborns who received a PNC check within 1 hour after delivery ➢ # of newborns who received a PNC check within 24 hours after delivery	# of newborns delivered in a facility or at home with a skilled attendant+
*Pre-discharge PNC*: # of newborns who received a PNC check before facility discharge	# of newborns delivered in a facility+
*PNC Content*: ➢ # of newborns whose umbilical cord was examined during a PNC check within 2 days of delivery ➢ # of newborns whose temperature was taken during a PNC check within 2 days of delivery ➢ # of mothers counseled on breastfeeding during a PNC check within 2 days of delivery ➢ # of mothers for whom breastfeeding was observed during a PNC check within 2 days of	# of newborns delivered in a facility or at home with a skilled attendant+
➢ delivery ➢ # of newborn/mother dyads who received all four PNC content components above	

*All indicators are limited to neonates born in the two years preceding the survey.

+ Data only collected in surveys using Demographic and Health Surveys Phase 7 (DHS7) questionnaires, generally 2016 and later.

**Indicator excludes data from Burkina Faso, Cambodia, Cameroon, Cote d’Ivoire, Mozambique, and Rwanda because these surveys do not collect data on this question.

As noted in Table 2, not all indicators are captured in all surveys analyzed. As a result of several rigorous measurement examinations^6,12,13^, the DHS, beginning with the DHS7 round implemented in approximately 2016, added to the questionnaire standardized indicators on whether neonates received immediate skin-to-skin contact, a postnatal care check before facility discharge, and four components of a postnatal care check within two days after delivery. These indicators are described further in Table 2. Because these indicators were only collected in recent DHS7 surveys, these data are only available in 21 of the countries analyzed, as indicated in Table 1. In addition, the DHS7 questionnaire changed the wording of survey questions regarding timing of PNC checks. Therefore, we have analyzed data on PNC timing from DHS7 surveys only, as results from previous DHS surveys are no longer comparable. This limitation is noted when presenting results only available for the smaller subset of countries that used DHS7 questionnaires.

We measure adherence to ENC standards among all births that are presumably attended by a health care worker: those delivered in public facilities, private facilities, or at home with a skilled attendant. We exclude home births without a skilled attendant, as there is already substantial evidence that these births result in poorer maternal and neonatal outcomes.^14^

To facilitate comparisons across sectors, we also limit some indicators to vaginal births, as we found that births in private facilities are more likely to be caesarian (C-section) births than those in public facilities (25% versus 11%) and are associated with longer health facility stays and lower levels of immediate breastfeeding and skin-to-skin contact. Please see S1, S2 and S3 Tables for results of these indicators by C-Section status.

We standardized delivery sources into four categories: 1) Public sector; 2) Private sector; 3) Home births attended by a skilled birth attendant (SBA); and 4) Home births unattended or attended by someone unskilled such as a traditional birth attendant, family member, or friend. Health facilities are disaggregated into public and private sector. We further disaggregate public facilities into hospitals and health centers (including clinics, health posts, and maternity centers) and private facilities into for-profit and non-for-profit facilities (the latter includes non-governmental and faith-based organizations).

### Data on sick neonates

To examine our second research question, we analyze data from mothers who gave birth in the month before the survey and therefore had a child less than 1 month old at the time of the survey. We examine the prevalence of fever among neonates in the two weeks preceding the survey, as fever is a symptom used to classify PSBI in neonates. Among neonates sick with fever, we examine the prevalence of out-of-home care seeking; and among neonates for whom treatment was sought, the source of that treatment. We compare the two-week fever prevalence, care seeking, and source patterns among neonates to those of older children.

Sources for sick neonatal care varied by country. We standardized these into three categories: 1) Public sector including public facilities (hospitals, clinics, health centers, and health posts) and community health workers; 2) Private sector including private facilities (hospitals, clinics, nongovernmental and faith-based organization) and private retail outlets (pharmacies, shops, and markets); and 3) Other including traditional healers, friends, and family.

### Ethics statement

We conducted a secondary analysis of DHS data, which is publicly-available, de-identified data that been reviewed by the ICF Institutional Review Board and typically by a review board in the host country, as well. Therefore, further ethical review was not necessary. The informed consent statement for each country survey is in each country’s respective final report. Additional details on the DHS’s ethical review procedures can be found here.

## Results

To answer the first research question, we analyzed DHS survey data from 289,729 mothers who gave birth in the two years preceding the survey. To answer the second research question, we analyzed DHS survey data from 8,231 mothers who gave birth in the month before the survey and therefore had a neonate less than one month old. Table 1 includes country and regional sample sizes for both research questions.

### Research question 1: What percentage of mothers and newborns receive essential newborn and postnatal care, and how does this vary by place of delivery?

Table 3 shows all research question 1 results by region and sector, including ranges across countries.

**Table 3:** ENC and PNC received by neonates born in the 2 years prior to survey by region and delivery source, DHS data from 45 countries.

	East and Southern Asia				East and Southern Africa				West and Central Africa				All countries analyzed			
	Range across countries analyzed						Range across countries analyzed				Range across countries analyzed				Range across countries analyzed	
	Pooled	p-value^1^	Min	Max	Pooled	p-value^1^	Min	Max	Pooled	p-value^1^	Min	Max	Pooled	p-value^1^	Min	Max
**Immediate Breastfeeding (++)**																
SBA Home Births	75.5	0.5	44.1	92.6	71.8	0.0	34.6	92.0	54.3	0.0	25.4	81.7	66.5	0.0	25.4	92.6
Public Facilities	76.5	0.0	35.8	87.1	79.6	0.0	37.7	95.5	61.6	0.0	25.5	82.1	71.6	0.0	25.5	95.5
Private Facilities	63.6	0.0	33.7	87.5	76.2	0.0	35.7	94.3	58.8	0.0	30.0	78.1	64.8	0.0	30.0	94.3
All	73.5		35.2	87.9	79.0		37.2	95.1	60.8		25.8	81.6	70.3		25.8	95.1
**Immediate Skin-to-Skin (** * **, ++)**																
SBA Home Births	53.1	0.0	6.0	75.5	27.6	0.0	0.0	60.2	20.0	0.0	11.3	49.6	37.8	0.0	0.0	75.5
Public Facilities	75.2	0.0	13.5	91.8	62.2	0.5	14.9	88.9	49.0	0.6	24.8	87.9	62.1	0.0	13.5	91.8
Private Facilities	59.3	0.0	10.1	86.0	63.6	0.0	24.2	83.9	50.1	0.0	13.1	88.0	59.0	0.0	10.1	88.0
All	68.7		11.2	86.5	61.2		15.2	87.0	47.0		19.5	87.3	60.1		11.2	87.3
**24+ hour facility stay (+, ++)**																
Public Facilities	66.3	0.0	14.6	95.4	62.8	0.0	31.3	91.2	53.7	0.0	9.3	86.9	59.9	0.0	9.3	95.4
Private Facilities	61.3		5.3	96.5	68.1		32.9	92.5	62.3		4.7	93.5	63.4		4.7	96.5
All	65.0		13.7	94.1	63.4		32.1	91.4	54.9		9.5	88.1	60.4		9.5	94.1
**PNC check within 1 hour of delivery (** * **)**																
SBA Home Births	12.6	0.0	0.4	51.3	5.8	0.0	0.7	16.5	7.7	0.0	1.6	22.4	10.4	0.0	0.4	51.3
Public Facilities	19.3	0.0	0.6	53.9	24.6	0.1	6.5	51.1	29.0	0.0	16.0	41.7	23.8	0.0	0.6	53.9
Private Facilities	29.0	0.0	1.0	52.9	26.9	0.0	7.5	52.9	32.8	0.0	28.4	48.8	28.9	0.0	1.0	52.9
All	21.2		0.6	51.5	24.3		5.9	50.5	28.0		15.3	41.6	23.8		0.6	51.5
**PNC check within 24 hours of delivery (** * **)**																
SBA Home Births	38.0	0.0	7.9	87.1	23.6	0.0	7.4	40.3	23.8	0.0	16.9	50.3	32.4	0.0	7.4	87.1
Public Facilities	66.8	0.0	11.9	93.1	66.1	0.0	33.9	88.4	68.5	0.0	58.8	79.2	66.9	0.0	11.9	93.1
Private Facilities	80.0	0.0	10.2	93.0	68.7	0.0	36.3	93.3	76.0	0.0	63.2	78.8	76.4	0.0	10.2	93.3
All	67.3		11.5	90.4	65.1		33.0	87.9	66.3		58.8	72.4	66.2		11.5	90.4
**Pre-discharge PNC check (** * **, +)**																
Public Facilities	85.9	0.0	75.3	96.7	71.5	0.0	35.2	94.6	73.7	0.0	63.3	80.9	76.0	0.0	35.2	96.7
Private Facilities	89.6		72.7	97.5	74.2		39.9	99.1	81.5		67.5	85.0	84.0		39.9	99.1
All	87.1		75.4	97.0	71.8		35.4	95.0	74.7		63.5	81.1	77.6		35.4	97.0
**PNC Content: Cord examined (** * **)**																
SBA Home Births	59.3	0.0	31.6	97.4	35.1	0.0	9.3	60.4	37.9	0.0	30.9	57.0	47.5	0.0	9.3	97.4
Public Facilities	69.1	0.0	37.4	89.8	54.7	0.1	5.7	86.9	43.9	0.0	32.6	50.0	56.1	0.0	5.7	89.8
Private Facilities	77.4	0.0	61.5	88.6	57.5	0.0	8.8	95.4	52.3	0.0	42.2	59.7	68.2	0.0	8.8	95.4
All	70.9		37.7	89.9	54.4		6.3	87.1	44.5		33.1	50.7	57.8		6.3	89.9
**PNC Content: Temperature taken (** * **)**																
SBA Home Births	50.2	0.0	25.1	96.2	28.4	0.0	6.2	45.8	32.8	0.0	22.6	53.2	40.1	0.0	6.2	96.2
Public Facilities	66.9	0.1	39.7	89.1	52.2	0.4	4.9	87.1	43.6	0.0	31.0	51.9	54.2	0.0	4.9	89.1
Private Facilities	69.3	0.0	59.3	88.1	53.4	0.0	7.1	95.6	54.5	0.0	42.5	58.3	62.7	0.0	7.1	95.6
All	66.4		39.7	89.2	51.6		5.3	87.3	44.1		31.1	52.8	55.0		5.3	89.2
**PNC Content: Breastfeeding counselling (** * **)**																
SBA Home Births	55.8	0.0	31.0	93.5	34.0	0.0	9.5	54.6	34.5	0.0	27.8	44.9	44.6	0.0	9.5	93.5
Public Facilities	66.9	0.1	48.7	85.4	59.8	0.5	6.2	85.3	42.2	0.0	35.2	49.6	57.6	0.0	6.2	85.4
Private Facilities	64.8	0.0	56.7	79.0	58.9	0.0	7.3	93.5	52.6	0.0	47.9	57.0	61.3	0.0	7.3	93.5
All	65.4		50.3	83.2	58.9		6.5	85.4	43.0		35.8	50.4	57.6		6.5	85.4
**PNC Content: Breastfeeding observed (** * **)**																
SBA Home Births	50.4	0.0	23.2	87.2	33.3	0.0	8.0	54.8	29.2	0.0	23.4	34.5	40.4	0.0	8.0	87.2
Public Facilities	61.4	0.0	28.3	76.1	55.7	0.9	5.5	81.5	34.0	0.0	21.9	42.0	52.1	0.9	5.5	81.5
Private Facilities	52.2	0.4	31.5	80.1	55.6	0.0	6.3	83.5	44.7	0.0	25.6	51.8	52.1	0.0	6.3	83.5
All	57.7		30.0	72.9	55.0		5.7	81.1	34.9		22.1	43.5	51.5		5.7	81.1
**PNC Content: All four components (** * **)**																
SBA Home Births	36.7	0.0	16.0	85.4	20.3	0.0	1.8	38.9	19.6	0.0	13.2	27.2	28.1	0.0	1.8	85.4
Public Facilities	47.0	0.0	22.7	61.4	39.8	0.8	2.3	72.6	25.1	0.0	20.4	30.8	38.3	0.0	2.3	72.6
Private Facilities	42.9	0.0	25.8	61.2	40.2	0.0	3.6	77.6	33.5	0.0	25.4	36.8	40.8	0.0	3.6	77.6
All	44.9		24.3	60.3	39.2		2.6	72.6	25.7		20.7	31.7	38.2		2.6	72.6

* Limited to DHS7 surveys.

+ Limited to facility births.

++ Limited to vaginal births.

#### Sources of delivery

In pooled analysis across the 45 countries analyzed, 57 percent of women delivered in a public facility (28 percent in a hospital and 29 percent in a clinic, health center, or maternity center), 12 percent in a private facility (10 percent in a for-profit facility and 2 percent in a non-for-profit facility), 4 percent at home with a SBA, and 26 percent at home without a SBA.

**Fig 1:**
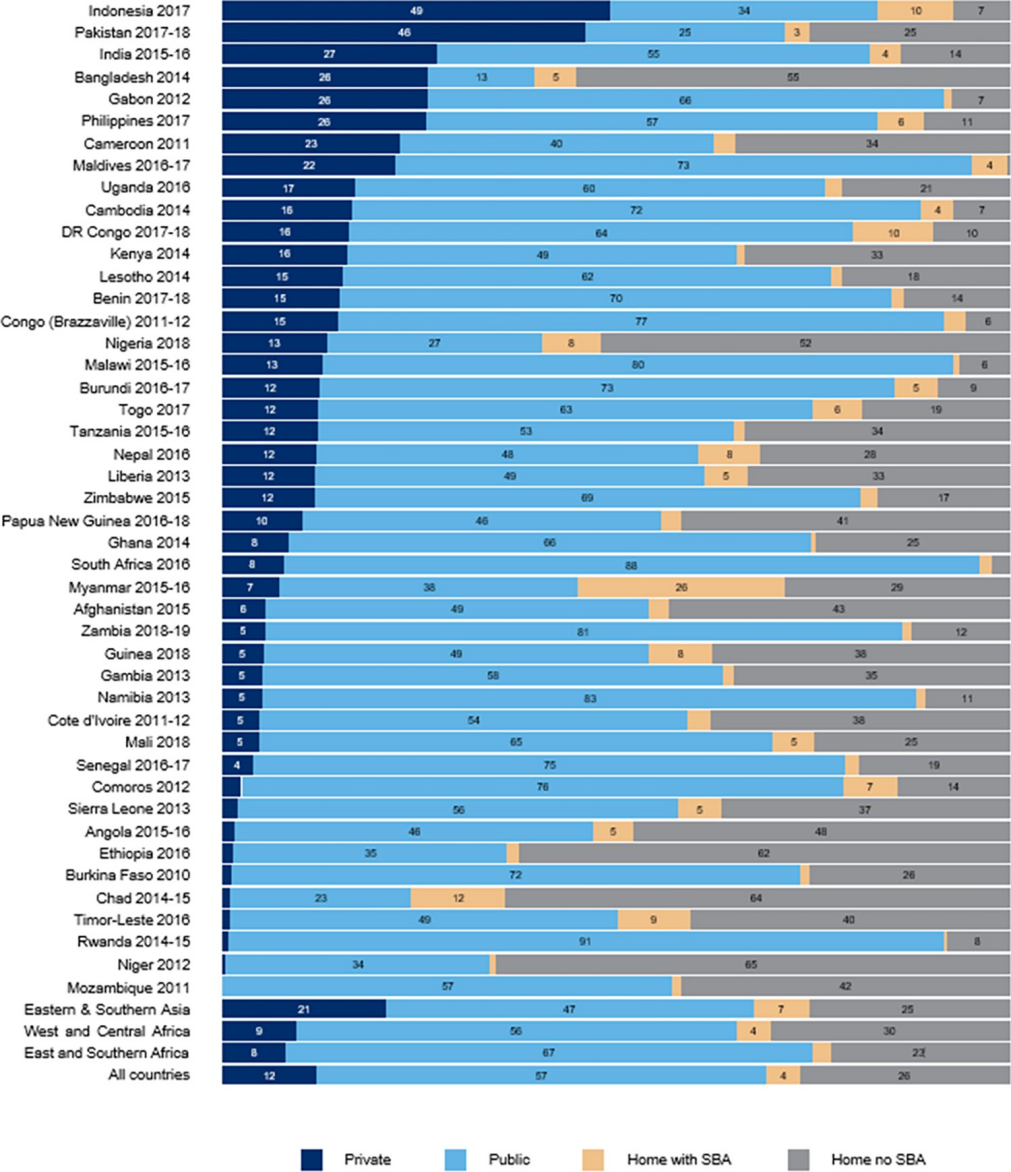
Distribution of delivery sources for the most recent birth in the past 2 years.

Delivery sources varied substantially by country (Fig 1). In Niger, Chad, Ethiopia, and Nigeria, more than 60 percent of births occurred at home without a skilled attendant. In South Africa, the Maldives, Malawi, Congo, Rwanda, and Gabon, conversely, more than 90 percent of births occurred within a public or private health facility. Indonesia and Pakistan had the highest levels of private sector use (49 and 46 percent, respectively). In more than half of countries analyzed—primarily in sub-Saharan Africa—the private sector was used by less than one in ten women for delivery.

#### Immediate breastfeeding

We examine breastfeeding within the first hour of life only among neonates born vaginally, as immediate breastfeeding rates were substantially lower among neonates delivered by C-section (46 percent versus 70 percent; see S1 Table for more detail). Among facility and home births with a SBA combined, 70 percent of neonates were breastfed within the first hour of life. This varies by region: 79 percent of neonates were immediately breastfed in East and Southern African countries analyzed compared to 73 percent of those in Asia and 61 percent of those in West and Central Africa. Mothers in several countries had immediate breastfeeding levels below 40 percent including those in Chad, Congo (Brazzaville), Pakistan, and Kenya. Mothers in Burundi and Rwanda had the highest levels of immediate breastfeeding (over 90 percent).

Immediate breastfeeding levels varied with place of delivery. Mothers were more likely to immediately breastfeed newborns delivered in public facilities (72 percent) than if they were born at home with a SBA or in a private facility (67 and 65 percent, respectively). Differences by source were largest in the Asian countries analyzed: 76 percent of newborns in public facilities were immediately breastfed compared to 64 percent of newborns in private facilities. In both sub-Saharan African regions, the lowest levels of breastfeeding within the first hour were seen among home births with SBAs.

#### Immediate skin-to-skin contact

Our analysis of immediate skin-to-skin contact is limited to neonates born vaginally, as skin-to-skin contact levels were substantially lower among women who had a C-section (36 percent versus 60 percent; see S2 Table). On average across the 21 countries with available data, 60 percent of neonates received skin-to-skin contact. Like immediate breastfeeding, countries in West and Central Africa had the lowest overall level of skin-to-skin contact at 47 percent. The levels in East and Southern Africa and Asia were higher at 61 and 69 percent, respectively. Mothers in Pakistan, Burundi, and Nigeria had the lowest skin-to-skin contact levels—all of which were below 20 percent.

Neonates born at home with a SBA were far less likely to receive immediate skin-to-skin contact (38 percent) than those born in a public (62 percent) or private facility (59 percent). This pattern was fairly consistent across regions but was particularly pronounced in West and Central African countries where 20 percent of children born at home with a SBA received skin-to-skin contact compared with approximately half of those born in a facility. There was one deviation to this pattern by region: in Asian countries, the level of skin-to-skin contact was lower among neonates born in the private sector (59 percent) and at home (53 percent) compared to those born in a public facility (75 percent).

#### 24+ hour facility stay

This indicator also excludes mothers and neonates delivered by C-section, as C-sections were associated with a longer health facility stay (94 percent versus 60 percent; see S3 Table).

The WHO recommends that all mother/baby dyads stay in health facilities for at least 24 hours after delivery. On average across all countries, 60 percent of mothers and newborns stayed in the facility for the recommended 24 hours or longer. In line with previous regional findings, 65 percent of mothers in Asian countries analyzed stayed in the facility for at least 24 hours compared to 63 percent of those in East and Southern Africa and 55 percent of those in West and Central Africa. In Guinea, Afghanistan, and Pakistan, mothers and newborns were extremely unlikely to stay in the facility for at least 24 hours (9, 14, and 16 percent, respectively). In contrast, 90 percent or more of mothers and newborns remained in the facility for 24+ hours in Malawi, Namibia, Myanmar, the Maldives, and the Philippines.

In the all-country pooled estimate, newborns delivered in private facilities were more likely than those delivered in public facilities to stay in the facility for 24+ hours (63 percent versus 60 percent). There are also sectoral differences in the percentage of mothers and newborns who stay in the facility for 24+ hours by region. In the East and Southern Africa region, the private sector had a higher level of adherence to the 24+ hour stay guideline than the public sector (68 versus 63 percent). In Asian countries analyzed, the reverse was true: 66 percent of mothers who delivered in the public sector stayed for 24+ hours compared to 61 percent of those in the private sector.

#### Timing of postnatal care visits

On average across all countries, 24 percent of newborns received a PNC check within the first hour of life and 66 percent received a PNC check within the first day (24 hours) of life. These indicators were consistent across the three regions examined.

By source, neonates born at home with a SBA were far less likely to receive a PNC check within their first 1 or 24 hours. Ten percent of mothers who gave birth at home with a SBA reported a PNC check for their newborn within one hour of delivery. In comparison, 24 and 29 percent of mothers who delivered in the public and private sectors, respectively, reported a PNC check within one hour of delivery.

Within 24 hours of birth, 32 percent of newborns delivered at home with a SBA received a PNC check. The level was substantially higher in the public (67 percent) and private sectors (76 percent). Among neonates born in a private facility, a PNC check within 24 hours was more common in Asian countries analyzed (80 percent) and least common in East and Southern African countries analyzed (69 percent). Among neonates born at home with a SBA, a PNC check within 24 hours was also much more common in the Asia region (38 percent) compared with both sub-Saharan Africa regions (24 percent). This indicator did not vary regionally for neonates born in the public sector.

#### Pre-discharge postnatal care check

On average across 21 countries in our analysis with available data, 65 percent of neonates who were delivered in a public or private facility received a pre-discharge postnatal check. This indicator was highest in Asian countries analyzed (74 percent), lower in West and Central African countries (67 percent), and lowest in East and Southern African countries (58 percent).

Neonates born in a private facility were more likely to receive a pre-discharge postnatal check than those born in a public facility (72 versus 64 percent). This disparity was most pronounced in West and Central Africa (65 percent private versus 56 percent public) and less so in East and Southern Africa (72 percent private and 66 percent public) and Asia (76 percent private and 72 percent public). Ethiopia had the lowest level of pre-discharge PNC checks across sectors– 40 percent in the private sector and 35 percent in the public sector. South Africa was an outlier compared to its regional average, with a high level of pre-discharge postnatal checks in private (99 percent) and public (95 percent) facilities.

#### Postnatal care content

This indicator examines if the neonate and mother received the following four components of postnatal care within two days after delivery: 1) neonate’s cord examined, 2) neonate’s temperature taken, 3) mother was counseled on breastfeeding, and 4) breastfeeding was observed. As above, data are only available for 21 of the countries included in this analysis. On average across these 21 countries, more than half of mothers and neonates received each check (51 percent for observed breastfeeding, 55 percent for check temperature, and 58 percent for breastfeeding counselling and cord examination), but fewer received all 4 PNC content components (38 percent). As with other ENC indicators, the proportion of neonates who received all 4 PNC components was lowest among analyzed countries in West and Central Africa (26 percent), higher among countries in East and Southern Africa (39 percent), and highest in Asia (45 percent).

Neonates born in a private facility were slightly more likely to receive all four PNC content components than those born in public facilities (38 versus 41 percent). Those born at home with a SBA were at a much greater disadvantage: just over one in four (28 percent) received all four components. This pattern concealed regional differences. In East and Southern African countries, the public and private sectors performed equally (40 percent). However, in Asia, neonates born in the public sector were slightly more likely to receive all four PNC content components (47 versus 43 percent). The converse is true in West and Central Africa, where one third of neonates born in the private sector compared to one-fourth of neonates born in the public sector received all four PNC content components. Neonates born at home with a SBA were much less likely to receive all four PNC components in the African countries analyzed (20 percent) than in the Asian countries analyzed (37 percent).

When examining each PNC content component individually, neonates born at home with a SBA were at a disadvantage compared to those born in the public or private sectors. The public and private sectors performed similarly for breastfeeding observation, but private facility health workers were more likely than public facility health workers to examine the neonate’s cord (68 versus 56 percent), take the neonate’s temperature (63 versus 54 percent), and counsel the mother on breastfeeding (61 versus 58 percent).

#### Differences within the public and private sectors

Across the ENC and PNC indicators, there were differences in adherence to global quality standards within the public sector by type of public facility: hospitals versus health centers, clinics, or posts. Neonates born in public hospitals were more likely than neonates born in public health centers to receive skin-to-skin contact (70 versus 57 percent), 4 PNC content components (48 versus 31 percent), a PNC visit before facility discharge (84 versus 70 percent), and to stay in the facility for >24 hours (68 versus 52 percent). These disparities were largely consistent across regions with one exception: in Asian countries analyzed, neonates born in a public health center were more likely than those born in a hospital to receive skin-to-skin contact (79 versus 73 percent). Interestingly, the pattern was also flipped in all regions for immediate breastfeeding: neonates born in a public health center were more likely to be immediately breastfed than those born in a public hospital (74 versus 68 percent).

Within the private sector, we compared ENC and PNC indicators between private non-for-profit versus private for-profit facilities. Few neonates (2 percent) were born in private not-for-profit facilities overall. On average across countries, neonates born in a not-for-profit private facility were more likely than those born in a for-profit facility to receive immediate skin-to-skin contact (68 versus 57 percent) or immediate breastfeeding (74 versus 63 percent). In the East and Southern African countries analyzed, neonates born in not-for-profit facilities were more likely to receive all 4 PNC content components (49 percent) than those in for-profit facilities (34 percent) but this difference was not apparent in the other regions. We saw the reverse pattern in the Asian region for the percentage who received a PNC check before facility discharge (91 percent for newborns in for-profit versus 75 percent for newborns delivered in not-for-profit facilities).

#### Variations in delivery, ENC, and PNC indicators by wealth quintile

Place of delivery varied substantially by wealth quintile. The wealthiest mothers were more likely than the poorest to deliver in a health facility (92 versus 51 percent). The wealthiest mothers were more likely than the poorest to deliver in a public facility (65 percent versus 46 percent) or a private facility (27 percent versus 5 percent). In contrast, the poorest were more likely to deliver at home without a SBA (44 versus 6 percent). Delivery at home with a SBA was low for both the poorest (5 percent) and wealthiest (3 percent).

**Fig 2:**
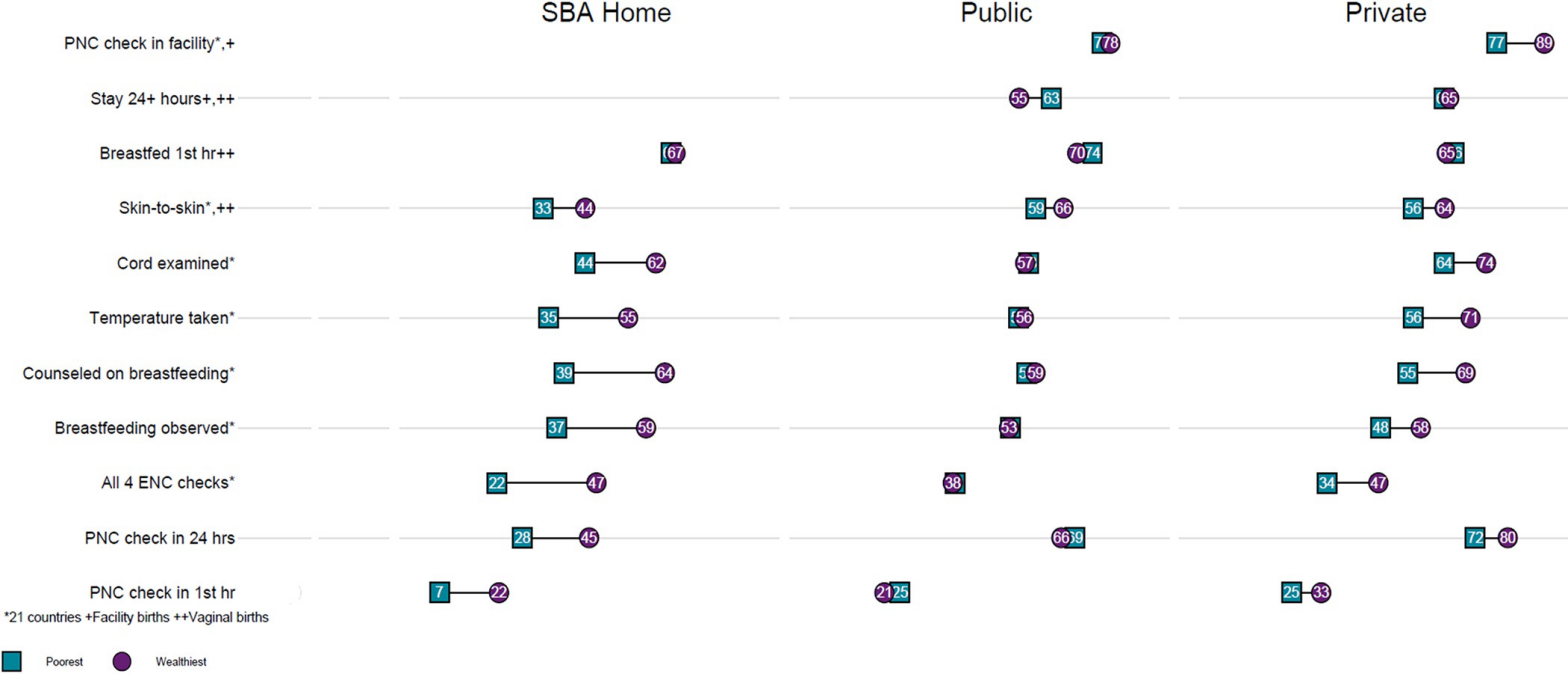
Receipt of ENC and PNC by newborns from the poorest and wealthiest households, by place of delivery.

When examining the ENC and PNC indicators by wealth quintile, Fig 2 shows that the disparities between newborns from the poorest and wealthiest households were largest among those delivered at home with a SBA, smaller among those delivered in private facilities, and smallest for newborns delivered in public sector facilities. There were different socioeconomic disparities within particular source categories for different indicators.

Among neonates born at home with a SBA, there were disparities of 10 or more percentage points between newborns from the wealthiest and poorest households for every indicator except for immediate breastfeeding. For example, receipt of all 4 PNC content components was more than twice as common among wealthiest than poorest families (47 versus 22 percent), a disparity of 25 percentage points.

Among neonates born in the public sector, there was a socioeconomic disparity in the percentage who received immediate skin-to-skin contact (59 percent poorest versus 66 percent wealthiest) and who stayed in the facility for >24 hours after birth (63 percent poorest versus 55 percent wealthiest). Note that the latter disparity in length of facility stay favored neonates from the poorest households rather than those from the wealthiest households. This latter disparity in length of facility stay was not seen for neonates born in the private sector.

Finally, in the private sector there were socioeconomic disparities in receipt of all four PNC content components (47 percent wealthiest versus 34 percent poorest), receipt of immediate skin-to-skin contact (64 percent wealthiest and 56 percent poorest), and receipt of a PNC check before facility discharge (89 percent wealthiest versus 77 percent poorest). Except for skin-to-skin contact, these disparities were not seen for neonates born in the public sector. Note that there were socioeconomic disparities in receipt of skin-to-skin contact for neonates across all three places of birth—home, public facilities, and private facilities.

#### Variations in delivery, ENC, and PNC indicators by maternal age

**Fig 3:**
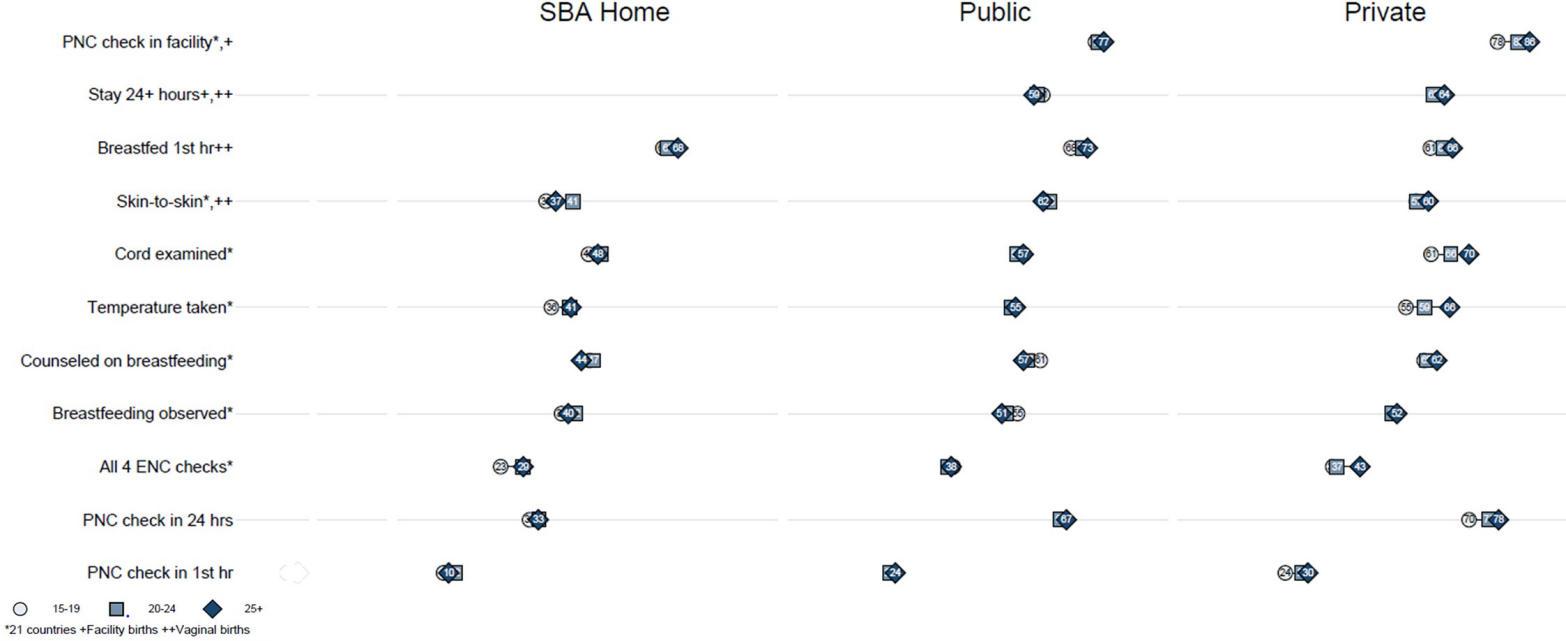
Receipt of PNC and ENC by maternal age and place of delivery.

When we examined delivery, PNC, and ENC indicators by maternal age categories (15–19 years, 20–24 years, and 25–49 years), there were fewer disparities than for socioeconomic groups. Levels of delivery at home and in the public and private sectors were very similar for mothers of all age groups (Fig 3). The primary disparities by maternal age were in the private sector between adolescent mothers 15–19 years and those in the oldest category from 25–49 years. These inequities in the private sector exist for five ENC and PNC indicators: the percent of newborns that received a PNC check within 1 and within 24 hours, who had their cord examined and temperature taken during a PNC check within two days after delivery, and that had all four measured PNC components completed. The largest private sector disparity was in the percent of newborns who had their temperature taken, with newborns of adolescent mothers 11 percentage points less likely to have received this critical component of PNC care compared with newborns of mothers age 25 or older (55 versus 66 percent, respectively). Patterns by maternal age are similar in non-for-profit facilities. Therefore, these disparities in the private sector can be attributed to disparities in for-profit private facilities. For example, the difference in taking the newborn’s temperature between mothers age 15–19 and 25 and older expands to 16 percentage points (50 versus 66 percent) among mothers who delivered in a private for-profit facility.

For neonates born in the public sector, the largest disparity by maternal age is a five percentage point difference in levels of immediate breastfeeding between mothers age 15–19 and 25+ (68 versus 73 percent). Among newborns delivered at home with a SBA, there are disparities of 5 or 6 percentage points for three indicators: 1) immediate skin-to-skin contact, 2) temperature taken, and 3) all four PNC content components completed.

Most of these disparities are much smaller than those seen between newborns from the poorest and wealthiest households who were delivered at home with a SBA.

### Research 1uestion 2: What are levels of and sources for care seeking for sick neonates?

Seeking treatment for sick neonates is key to enhancing neonatal health and survival. We investigate the care-seeking levels and sources for care of newborns sick with fever as a tracer indicator for PSBI in neonates. We begin by examining the prevalence of fever in newborns compared to older infants and children.

#### Neonatal prevalence of fever

On average across the 45 countries analyzed, 4 percent of neonates experienced fever in the 2 weeks prior to the survey (Table 4). The two-week prevalence of fever was consistent across the three regions analyzed, but there was substantial variation at the country level. The countries with the highest levels of fever among neonates were Lesotho (10 percent) and Pakistan (14 percent).

**Table 4:** Fever prevalence, care-seeking level, and sources of care by child’s age and region.

			Among children with fever		Among children for whom care was sought outside the home					
Age and region	Two-week fever prevalence	Number of children	Care sought outside the home	Number of children with fever	Public Facilities	Public CHWs	Private Facilities	Private Retail Outlets	Other source	Number of children for whom care was sought outside the home
**0–29 days**										
East and Southern Asia	3.9	3,086	65.5	121	20.6	1.6	43.6	34.2	0.2	75
East and Southern Africa	4.2	2,178	45.7	99	63.3	0.0	22.1	13.6	1.0	41
West and Central Asia	4.5	2,822	46.5	150	76.2	0.0	1.5	20.6	2.8	65
All countries analyzed	4.2	8,086	51.1	370	53.8	0.5	21.8	22.9	1.4	181
**30–59 days**										
East and Southern Asia	10.6	5,937	57.5	485	37.7	1.2	43.7	21.0	4.1	318
East and Southern Africa	9.6	2,694	55.7	275	66.2	3.1	19.1	9.2	4.1	153
West and Central Asia	8.5	4,076	52.3	374	60.2	0.0	10.7	26.8	7.1	188
All countries analyzed	9.4	12,707	54.9	1,134	55.4	1.4	23.5	19.2	5.2	659
**2–11 months**										
East and Southern Asia	26.1	64,099	75.7	12,146	34.4	1.6	46.3	19.4	3.3	9,466
East and Southern Africa	22.8	26,645	67.1	6,403	72.0	1.7	16.8	10.6	2.0	4,451
West and Central Asia	22.4	40,139	64.5	9,308	59.2	0.6	9.7	28.5	6.2	5,843
All countries analyzed	23.5	130,883	68.5	27,857	55.5	1.3	23.4	20.0	3.9	19,760
**12–23 months**										
East and Southern Asia	27.5	74,412	76.6	14,957	37.3	1.2	42.4	22.5	2.5	11,662
East and Southern Africa	24.6	31,008	67.5	8,014	70.4	2.0	17.2	10.7	2.7	5,593
West and Central Asia	25.2	44,180	65.8	11,588	57.1	0.9	11.3	28.8	5.7	7,470
All countries analyzed	25.6	149,600	69.4	34,559	55.1	1.4	22.9	21.1	3.8	24,725
**24–59 months**										
East and Southern Asia	21.0	228,422	76.9	32,173	36.1	1.3	41.3	23.9	2.7	24,524
East and Southern Africa	18.1	89,565	66.2	17,192	65.9	2.5	17.4	14.1	2.5	11,773
West and Central Asia	17.4	133,437	63.7	24,090	53.5	0.8	11.6	31.1	6.6	15,024
All countries analyzed	18.6	451,424	68.6	73,455	51.4	1.5	23.6	23.4	4.0	51,321

Shaded cells are based on 25–49 unweighted cases and should be interpreted with caution.

**Fig 4:**
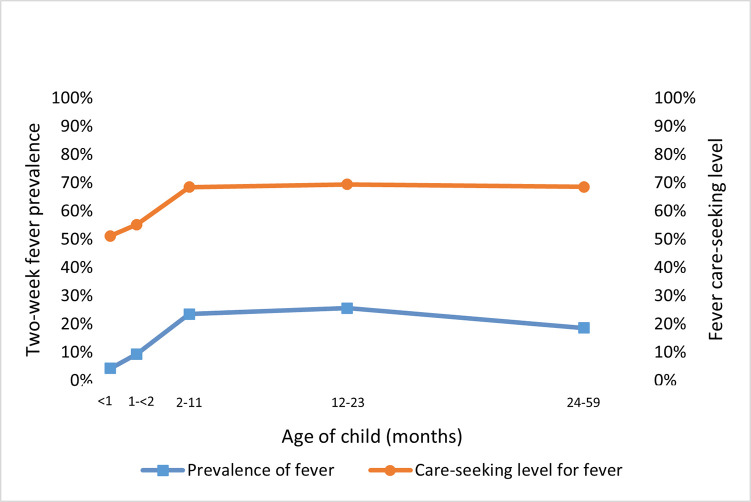
Illness prevalence and care seeking level by age group.

The prevalence of fever increased once children pass neonatal age, as shown in Fig 4. Among young infants 30–59 days old, the prevalence of fever was 9 percent, and for infants 2 months to less than a year old, fever prevalence increased to 23 percent. The two-week fever prevalence was only slightly higher (26 percent) among children 12 to 23 months old. Interestingly, among children 24 to 59 months old, the prevalence decreased to 19 percent. These patterns for each of the older age groups was consistent across the three regions analyzed (Table 4).

#### Care seeking outside the home for neonates with fever

Among caregivers of neonates sick with fever, just over half (51 percent) sought advice or treatment outside of the home. Caregivers in Asian countries analyzed were more likely to seek care for their sick neonates (66 percent) than those in West and Central African countries (47 percent) or those in East and Southern African countries analyzed (46 percent).

Care seeking for fever was higher for post-neonatal infants than for neonates. As shown in Fig 4, care was sought for 55 percent of sick young infants 30–59 days old and increased to 69 percent for infants 2–11 months old. After this age group, the care-seeking level remained stable at 69 percent for sick children 12–23 months old and children 24–59 months old. Across these older age groups, the care-seeking level was consistently higher in Asian countries than in sub-Saharan African countries analyzed by about 10 percentage points.

#### Sources of care for newborns with fever

Among caregivers who sought advice or treatment outside the home for their neonates with fever, 54 percent used public facilities, 45 percent used private sources (22 percent private facilities, 23 percent private retail outlets), and 1 percent went to other sources such as family or friends. Less than one percent of caregivers reported using community health workers for sick neonatal care.

**Fig 5:**
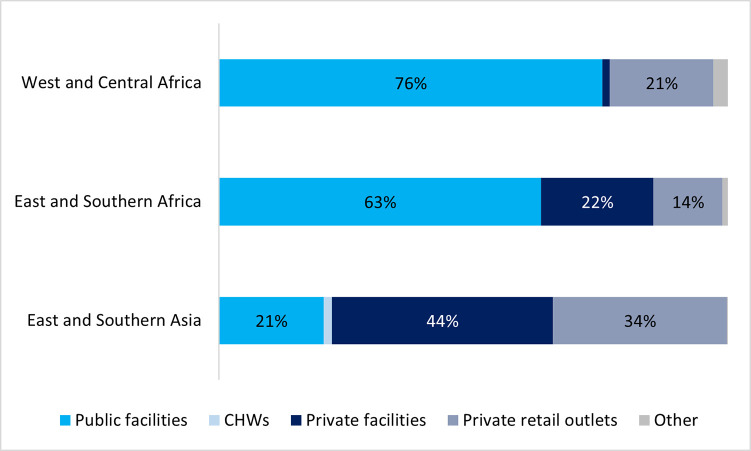
Sources of care for neonates by region, among neonates sick with fever whose caregivers sought treatment or advice outside the home.

These source patterns differed by region, shown in Fig 5. Use of public facilities was most common in West and Central Africa (76 percent) followed closely by high use in East and Southern African countries (63 percent). However, just over one-fifth (21 percent) of caregivers in Asian countries analyzed went to public facilities for their sick neonates. Instead, 78 percent of Asian caregivers went to private sources: 44 percent to private facilities and 34 percent to private retail outlets. Care-seeking in private facilities was less common in East and Southern African countries analyzed (22 percent) and extremely infrequent in West and Central Africa (1 percent). Use of private retail outlets (pharmacies and shops) was somewhat more similar across the three regions, ranging from 14 percent in East and Southern Africa to 21 percent in West and Central Africa and 34 percent in Asia.

**Fig 6:**
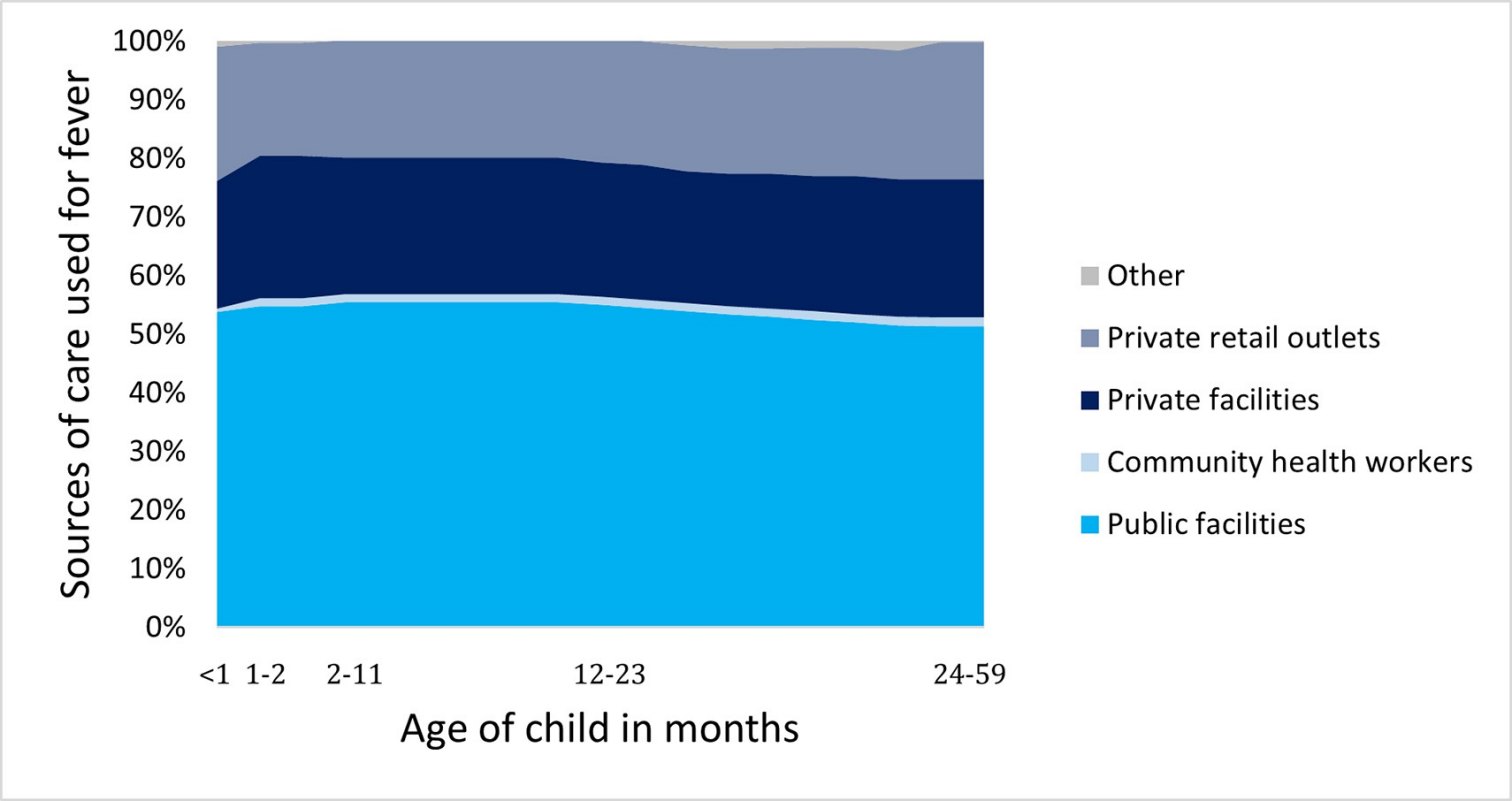
Sources for care by age group, among children in each age group sick with fever whose caregivers sought treatment or advice outside the home.

Though the two-week fever prevalence changed by age, Fig 6 shows that the sources parents used did not vary substantially as children get older, demonstrating that caregivers largely relied on the same sources regardless of their children’s age.

#### Variations in sick neonatal care by wealth quintile

**Fig 7:**
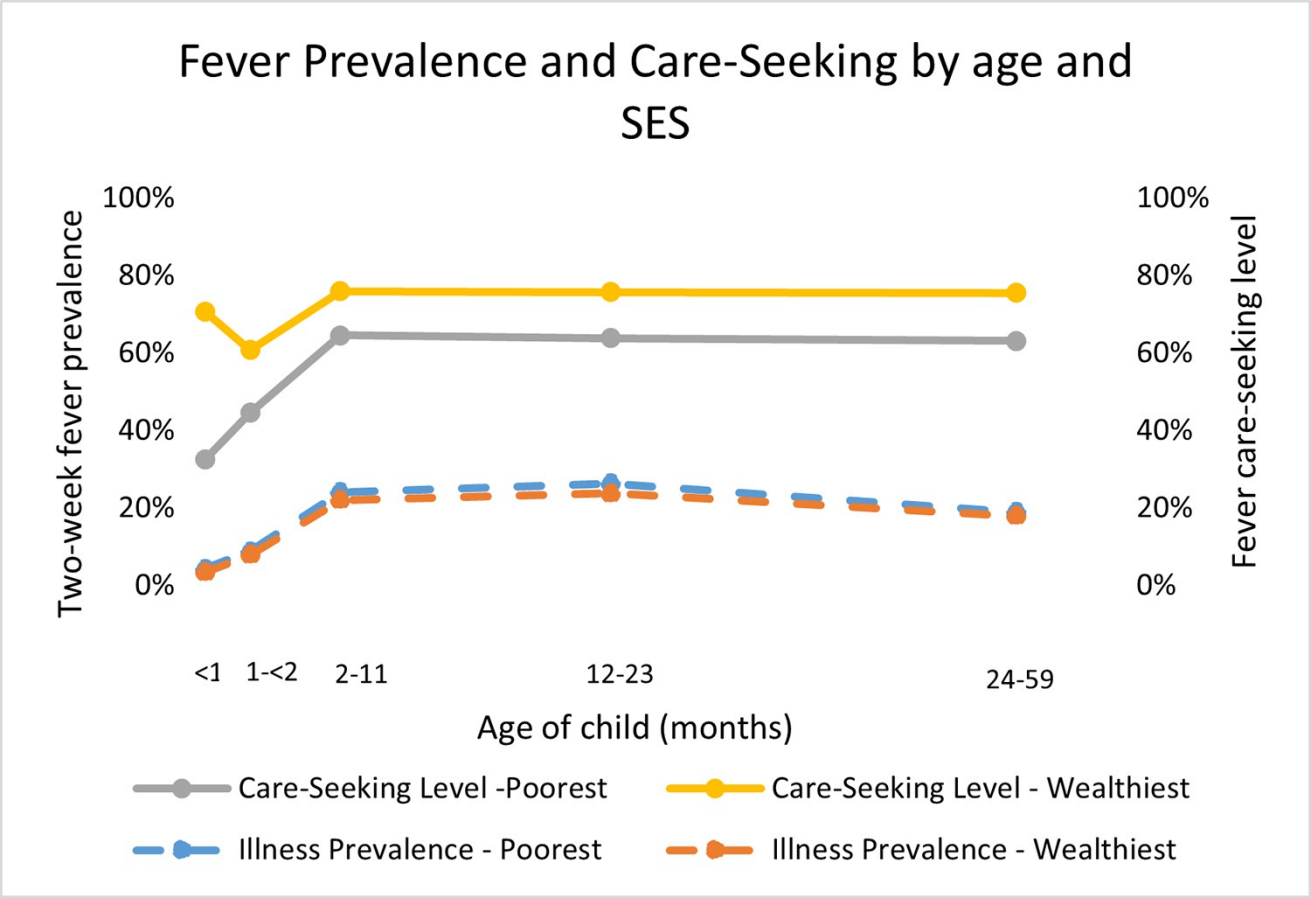
Fever prevalence and care-seeking by age and socioeconomic status.

Among neonates and on average across countries, there was not a socioeconomic disparity in prevalence of fever (Table 5). In contrast, there was a stark socioeconomic disparity in care-seeking levels for neonates with fever: just 32 percent of neonates from the poorest families received advice or treatment outside the home compared with 71 percent of neonates from the wealthiest families. Note that when the number of neonates with fever is disaggregated by wealth quintile, sample sizes are small (n = 25–49) and should be interpreted with caution. The care-seeking level among the poorest families increased with the child’s age to 45 percent among the poorest families with young infants 30–59 days old and increased further to 65 percent among the poorest families with infants 2–11 months old (Fig 7). This level remained consistent for children 12–59 months from the poorest families. The socioeconomic disparity in care seeking was greatest for neonates and narrower for older children.

**Table 5:** Fever prevalence, care-seeking level, and sources of care by socioeconomic status within each age group.

	0–29 days			30–59 days			2–11 months			12–23 months			24–59 months		
	Q1	Q5	p-value	Q1	Q5	p-value	Q1	Q5	p-value	Q1	Q5	p-value	Q1	Q5	p-value
**Fever prevalence**	4.3	3.4	0.43	8.9	7.9	0.48	24.0	22.0	0.01	26.3	23.7	0.00	18.9	17.9	0.01
Number of children	2,187	1,117		3,173	1,873		32,927	19,483		37,851	22,131		117,184	64,941	
**Care-seeking level**	32.5	70.7	0.00	45.0	61.6	0.04	64.6	75.9	0.00	63.8	75.7	0.00	63.1	75.5	0.00
Number of children with fever	111	39		280	130		7,160	3,819		8,963	4,676		19,272	9,958	
**Sources for sick child care**															
Public Sources	27.0	68.8	0.00	50.2	46.0	0.49	62.8	47.9	0.02	61.9	46.4	0.01	58.2	41.1	0.00
Private Sources	66.5	31.2	0.96	47.7	57.7	0.06	35.5	53.4	0.00	36.2	56.0	0.00	39.9	61.1	0.00
Number of children for whom care was sought outside the home	35	30		136	85		4,786	2,998		5,997	3,632		12,514	7,707	

Q1 refers to the poorest 20% and Q5 refers to the wealthiest 20% of households in each country.

p-values from logistic regressions to test for differences in proportions between children in Q1 and Q5`.

Shaded cells are based on 25–49 unweighted cases and should be interpreted with caution.

When comparing care-seeking sources for neonates from the poorest and wealthiest households, the biggest difference was in use of public sources. Caregivers from the wealthiest households were more likely than those from the poorest households to use public sources for sick neonates (69 versus 27 percent). Sixty six percent of caregivers from the poorest households and 31 percent from the wealthiest went to private sector sources for their sick neonates. While use of private sector sources is higher among the poorest than wealthiest families, this difference is not statistically significant, and these results should be interpreted with caution as the sample size is less than 50 observations.

## Discussion

Delivery with a skilled attendant, adherence to ENC and PNC standards, and immediate quality treatment of sick neonates are all essential components of high-quality neonatal care and are critical to improve maternal and child survival. This study highlights some successes in these matters and many areas for improvement in the delivery of quality of neonatal care. This study provides novel insights into how ENC, PNC, and fever care seeking for neonates vary across sectors.

In pooled analysis across the 45 countries analyzed, more than 1 in 4 deliveries occurred at home without a skilled attendant, making it difficult to obtain many ENC and PNC components as well as basic life-saving and emergency care. Among newborns delivered by a healthcare provider, we can glean information regarding quality from levels of adherence to ENC and PNC standards. Adherence to ENC and PNC standards was universally higher for births in health facilities compared to home births with a SBA with the notable exception of immediate breastfeeding. Among neonates born in public facilities, those born in a hospital were generally more likely to receive ENC and PNC than neonates born in a public health clinic, center, or post. There are positive signs in relatively high levels of adherence to immediate breastfeeding and pre-discharge postnatal care visits for facility-born neonates. However, levels of adherence to recommended PNC content were universally low, on average, as was the level of PNC checks within one hour of delivery. The public sector had significantly higher levels for immediate breastfeeding and skin-to-skin contact, while the private sector has higher levels of adherence to all other ENC and PNC indicators, with the exception of observing breastfeeding during the PNC check, which was similar in both sectors. This finding differs from studies in Ghana and Eastern Uganda that found that public sector providers performed better than private providers in provision of essential newborn care [21,22].

While the proportion of mothers who delivered in a health facility is lower than ideal (69 percent), it is higher than the proportion who seek care outside of their home when their neonates experienced fever (51 percent). When mothers or caregivers do leave their homes for delivery or care seeking, the private sector played a larger role for sick neonatal care (45 percent) than for delivery (18 percent).

Across nearly all indicators examined—comprehensive of delivery, ENC, PNC, and sick neonatal care—there are substantial socioeconomic disparities. Neonates from the poorest households were less likely to be born in a facility and were far less likely to receive care outside the home if they experienced fever. Among neonates born at home with a skilled attendant, those from the poorest households were less likely than those from the wealthiest to receive essential newborn and postnatal care, which aligns with findings from a 2021 study on co-coverage and equity of newborn care [24]. Socioeconomic disparities were largest among neonates born at home with a SBA and smallest among neonates born in the public sector. In many low- and middle-income country settings, cultural norms dictate that mothers and newborns should remain in the home, which could be a barrier to receiving ENC and PNC for mothers who delivered at home [25]. Notably, when SBAs were attending to newborns from the wealthiest families, they were capable of providing care at the same or higher levels than in the public sector. In the private sector, adherence to ENC and PNC standards was relatively high for mothers and newborns from the wealthiest households, indicating that adhering to recommended guidelines is achievable in particular settings. The remaining challenge is to achieve these relatively higher levels of care in all settings and narrow socioeconomic gaps across all sectors, particularly for neonates born at home with a SBA.

Conversely, there were far fewer inequities by maternal age, largely indicating that adolescent and other young mothers do not receive less or poorer-quality care for their newborns as compared to older mothers. This aligns with the findings from a study of 16 low- and middle-income countries found that maternal age was a significant predictor of newborn care only in Senegal and was non-significant in all other countries [24]. However, we found several disparities between the youngest and oldest maternal age groups in the private for-profit sector. In these facilities, adolescent mothers and their newborns received inferior care for some ENC and PNC indicators, and it is critical that these for-profit private sector gaps are addressed to ensure that newborns of young mothers receive the best quality of care.

### Limitations

It is challenging to ascertain quality solely from care-seeking data, as the DHS does not collect information on the provider-caregiver interaction. In addition, fever is a non-specific proxy for PSBI and could be representative of other causes of post-neonatal morbidity and mortality. Further investigation, particularly through the analysis of routine facility data and health facility assessments, is warranted to better understand the quality of sick young infant care. Finally, the quality of the findings presented is dependent on the quality of DHS data. The DHS program has rigorous quality assurance standards and aims to collect standardized data across countries through standard questionnaires and data collection training techniques. However, there may be differences in how the survey is administered across countries, which could result in modest differences in question interpretation and results.

## Conclusion

Adherence to essential newborn and postnatal care is low across the public and private sectors and especially among home births with skilled attendants, demonstrating great need for improvements in quality of neonatal care. Adherence to global ENC and PNC standards is consistently higher in health facilities compared to home births with SBAs. In addition, adherence to these standards is higher in private facilities than public facilities, with the exception of immediate breastfeeding and skin-to-skin contact, which is more common among mothers and newborns in public facilities. Socioeconomic disparities persist in access to skilled delivery, adherence to ENC and PNC—particularly among newborns delivered at home with a skilled attendant, and in neonatal care seeking for fever. While there have been positive trends in reducing socioeconomic disparities in neonatal mortality,^15^ closing inequities in neonatal care and care seeking and ensuring that all families, including the poorest, can access high quality maternal and newborn care is crucial to continue positive trends and further accelerate reductions in neonatal and child mortality.

## Supporting information

S1 Table Percentage of newborns who received immediate skin-to-skin contact by C-section status (includes all facility and attended home births; limited to countries with a DHS7 survey). (XLSX)

S2 Table Percentage of newborns who received immediate breastfeeding by C-section status (includes all facility and attended home births). (XLSX)

S3 Table Percentage of newborns who stayed in the facility for at least 24 hours after delivery by C-section status (includes all facility births). (XLSX)
